# Supplementary material for: Structure of a seeded palladium nanoparticle and its dynamics during the hydride phase transformation
Source: Commun Chem. 2021 May 11;4:64. doi: 10.1038/s42004-021-00500-7 (PMC9814609; doi:10.1038/s42004-021-00500-7)
Supplement: Supplementary file 1 — Supplementary Information [file 42004_2021_500_MOESM1_ESM.pdf]

Structure of a seeded palladium nanoparticle and its dynamics during the hydride phase transformation

Ana F. Suzana[1,*], Longlong Wu[1], Tadesse A. Assefa[1], Benjamin P. Williams[2], Ross Harder[3], Wonsuk Cha[3], Chun-Hong Kuo[4], Chia-Kuang Tsung[2] and Ian K. Robinson[1,5,*]

[1] *Condensed Matter Physics and Materials Science Department, Brookhaven National Laboratory, Upton, NY 11793, USA*

[2] *Department of Chemistry, Merkert Chemistry Center, Boston College, Chestnut Hill, MA 02467, USA*

[3] *Advanced Photon Source, Argonne National Laboratory, Lemont, IL 60439, USA*

[4] *Institute of Chemistry, Academia Sinica, Taipei 11529, Taiwan*

[5] *London Centre for Nanotechnology, University College London, London WC1E 6BT, UK*

Supplementary information

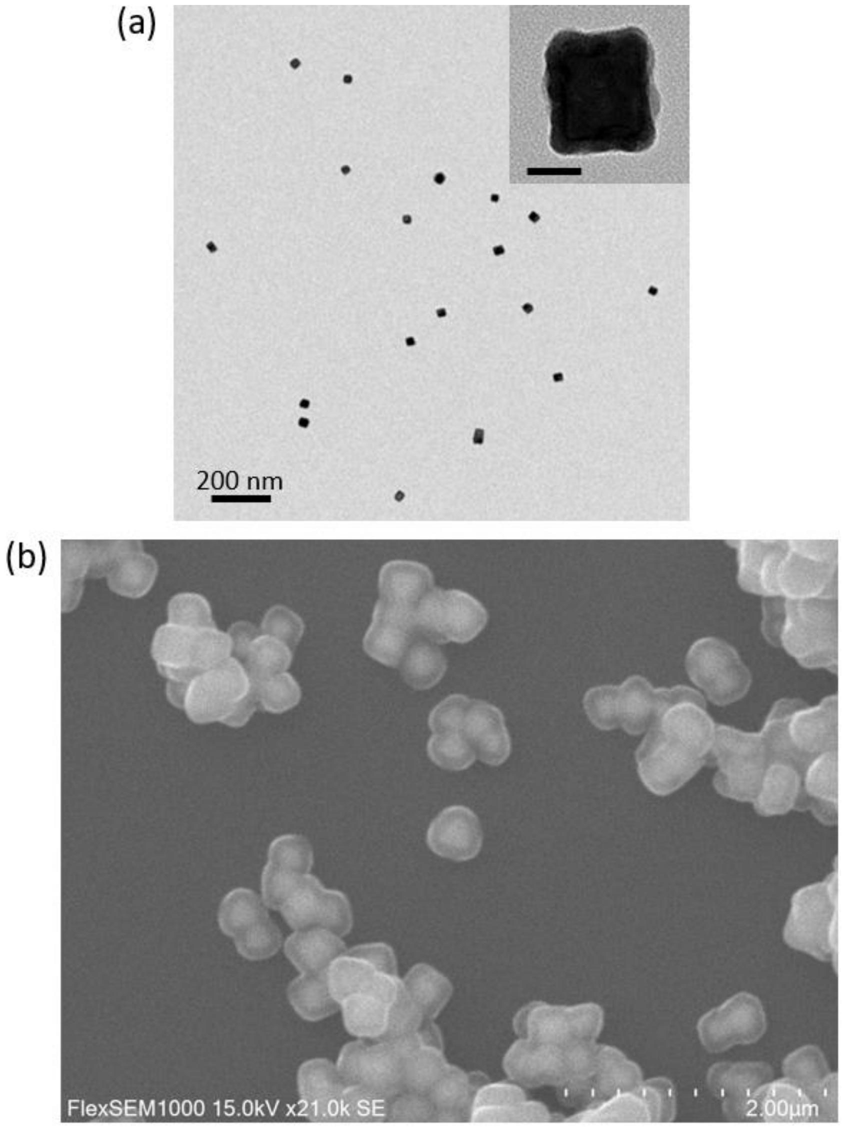

Supplementary Figure 1. Electron microscopy images of the seed precursor and the final nanocrystal. (a) Bright-field TEM images of the Pd seeds. The scale bar in the inset panel is 20 nm. (b) SEM image of the TiO_2-coated Pd nanocrystals studied in the BCDI experiment.

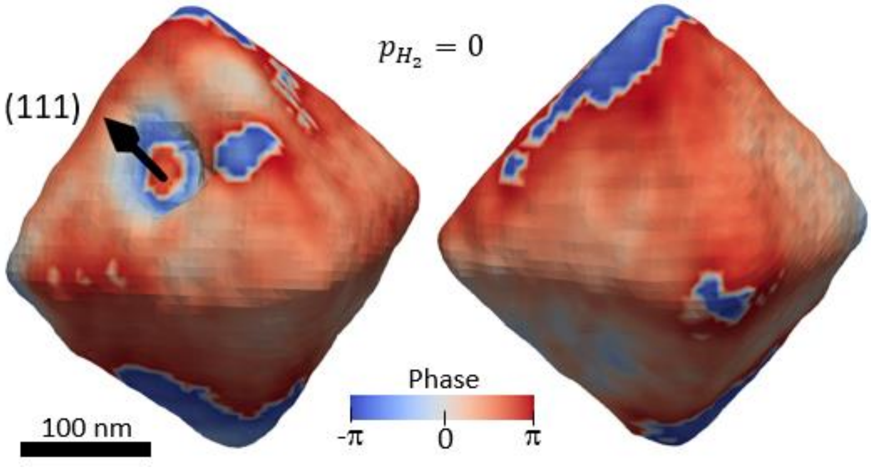

Supplementary Figure 2. The original wrapped real phase for the nanoparticle measured under pristine conditions. The [111] direction is shown as the black arrow.

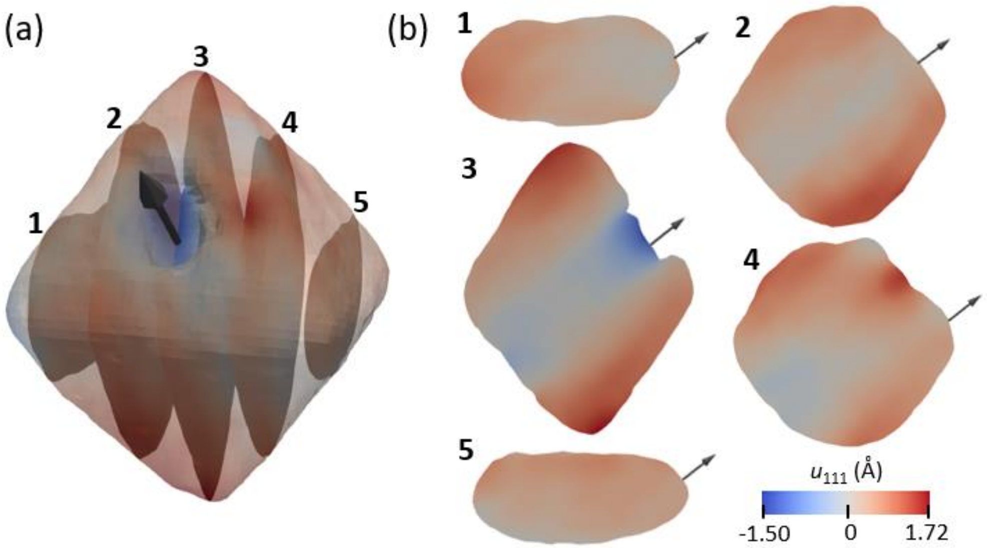

Supplementary Figure 3. Displacement distribution map shown as cross-section views for the nanoparticle measured under pristine conditions. (a) Spatial positions of the cross-sections (1-5) shown in (b). The volume particle is displayed as a semi-transparent isosurface. The [111] direction is shown as the black arrow in (a) and in all cross-sections in (b).

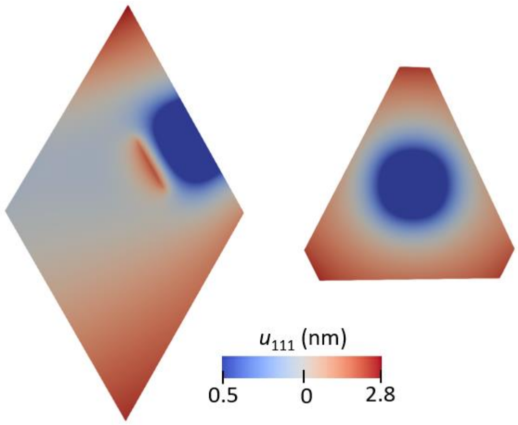

Supplementary Figure 4. Finite element model results using a symmetric octahedron. Cross-section of the model (left) taken in the x direction, corresponding to the direction of the Q vector in the experimental data. Cross-sectional view (right) taken in the same position as cross-section number 1 shown in Fig. 4.

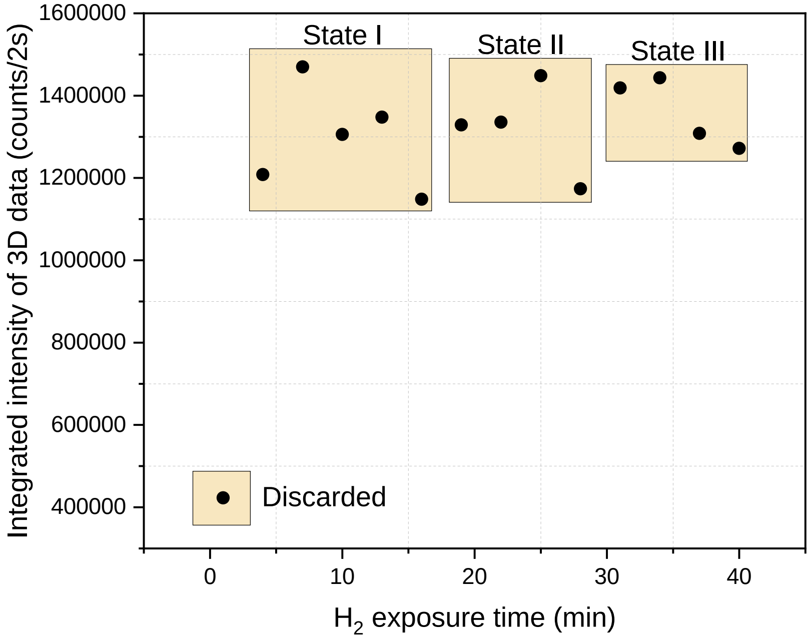

Supplementary Figure 5. Plot showing the integrated intensity of the 3D data as a function of hydrogen exposure time. The first scan was discarded and the following ones (from time = 4-40 min), representing states I, II and III, were averaged in groups to improve statistics.

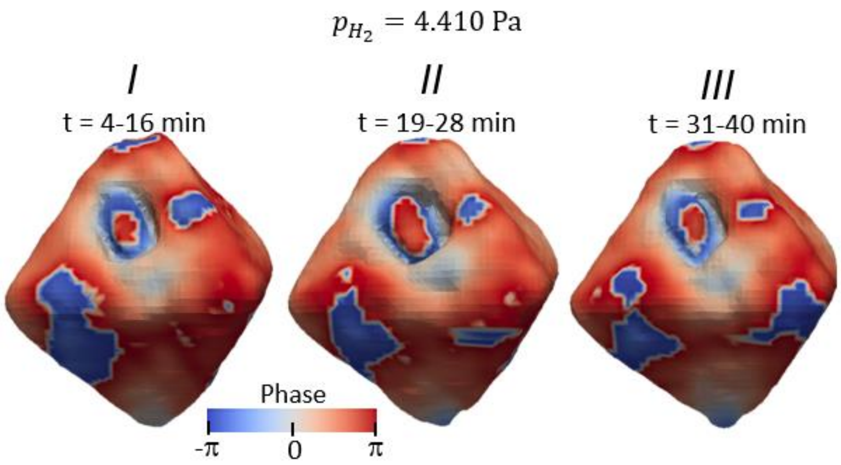

Supplementary Figure 6. Reconstructed wrapped phases for states *I-III* shown in Fig. 5. The nanoparticle was measured under a flux of hydrogen ($p_{H_2} = 4.410\ Pa$) through the sample to induce the α-β phase transformation.
